# Computational studies on the catalytic potential of the double active site for enzyme engineering

**DOI:** 10.1038/s41598-024-60824-x

**Published:** 2024-08-02

**Authors:** Naveen Banchallihundi Krishna, Lalitha Roopa, R. Pravin Kumar, Gopenath T S

**Affiliations:** 1 Present Address: Department of Computational Biology and AI, Kcat Enzymatic Private Limited, #16, Ramakrishnappa Road, Cox Town, Bangalore, 560005 India; 2 Department of Biotechnology and Bioinformatics, JSS Academy of Higher Education and Research, Mysuru, 570015 India

**Keywords:** Biotechnology, Computational biology and bioinformatics, Structural biology

## Abstract

Proteins possessing double active sites have the potential to revolutionise enzyme design strategies. This study extensively explored an enzyme that contains both a natural active site (NAS) and an engineered active site (EAS), focusing on understanding its structural and functional properties. Metadynamics simulations were employed to investigate how substrates interacted with their respective active sites. The results revealed that both the NAS and EAS exhibited similar minimum energy states, indicating comparable binding affinities. However, it became apparent that the EAS had a weaker binding site for the substrate due to its smaller pocket and constrained conformation. Interestingly, the EAS also displayed dynamic behaviour, with the substrate observed to move outside the pocket, suggesting the possibility of substrate translocation. To gain further insights, steered molecular dynamics (SMD) simulations were conducted to study the conformational changes of the substrate and its interactions with catalytic residues. Notably, the substrate adopted distinct conformations, including near-attack conformations, in both the EAS and NAS. Nevertheless, the NAS demonstrated superior binding minima for the substrate compared to the EAS, reinforcing the observation that the engineered active site was less favourable for substrate binding due to its limitations. The QM/MM (Quantum mechanics and molecular mechanics) analyses highlight the energy disparity between NAS and EAS. Specifically, EAS exhibited elevated energy levels due to its engineered active site being located on the surface. This positioning exposes the substrate to solvents and water molecules, adding to the energy challenge. Consequently, the engineered enzyme did not provide a significant advantage in substrate binding over the single active site protein. Further, the investigation of internal channels and tunnels within the protein shed light on the pathways facilitating transport between the two active sites. By unravelling the complex dynamics and functional characteristics of this double-active site protein, this study offers valuable insights into novel strategies of enzyme engineering. These findings establish a solid foundation for future research endeavours aimed at harnessing the potential of double-active site proteins in diverse biotechnological applications.

## Introduction

Lipase, due to its excellent substrate selectivity, regioselectivity and stereoselectivity, are used in numerous industrial bioreactions and bioprocesses^1^. Lipases can be produced from numerous microorganisms, including bacteria, fungi, and plants and are used for various processes like hydrolysis, esterification, transesterification, interesterification, alcoholyses, aminolysis, and thiolysis. It is utilised in various industries, like agrochemicals, cosmetics and flavours, pharmaceuticals, biodiesel manufacturing, food and beverages, detergents, textile, and paper industries^2–6^. Engineered lipases such as Novozyme 435 from Candida antarctica type B (CAL-B), Lipozyme Tl IM, Lipozyme RM IM etc... are examples that demonstrate how resilient these enzymes are even in harsh industrial reaction conditions^7^.

**Figure 1:**
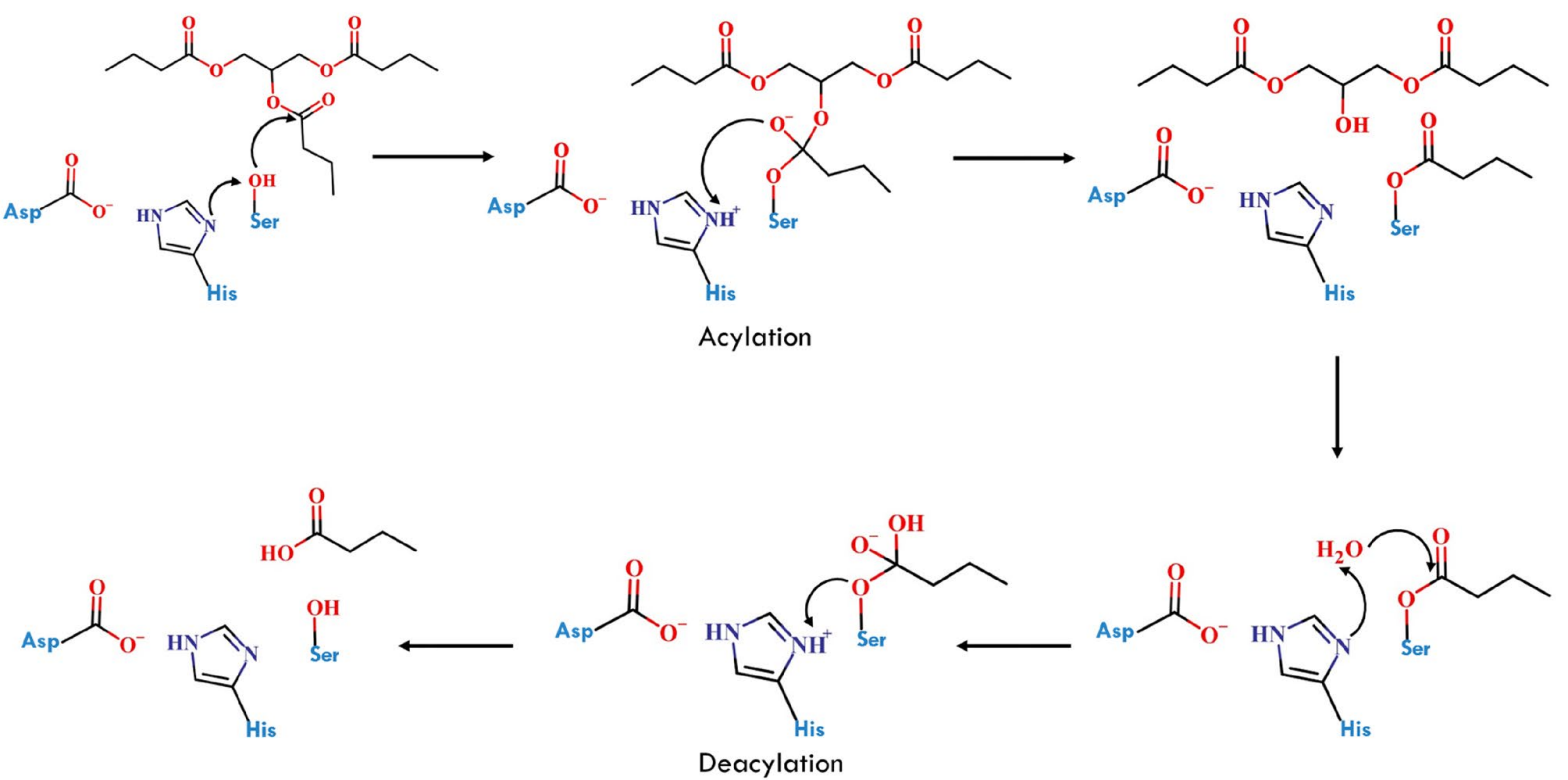
The hydrolysis reaction mechanism performed by lipase enzyme with the help of water molecules. The reaction starts with the active site reconfiguration where Aspartic acid abstracts proton from the Histidine simultaneously Histidine abstracts a proton from Serine. Nucleophilic attack on the carboxyl carbon of the substrate by Serine leads to the formation of an acyl-enzyme intermediate. The water molecule attacks on the carbonyl carbon of the substrate which breaks the bond between Serine and carbonyl carbon.

The lipase structurally belongs to the hydrolase fold family, which has special structural features including a flexible pocket that can accommodate a range of substrate sizes, a hydrophobic sheet at the centre of the enzyme, and two layers of helixes around it^8,9^. Esterase, hydrolase, and many other members of the lipase family have comparable structural characteristics. All lipase holds similar architecture of the catalytic site and reaction mechanisms which include Serine as important catalytic residue needed for the reaction. Using Serine and other catalytic residues along with a water molecule, the general hydrolase (lipase) enzymes carry out the hydrolysis reaction^10^. The reaction process can be explained in two steps which are Acylation and Deacylation^11^. The Acylation step in the reaction mechanism is initiated by the catalytic Histidine which protonates catalytic Serine (Fig. 1). The nucleophilic attack by the oxygen of Serine on carboxyl carbon of the substrate leads to the formation of the acyl-enzyme intermediate^12^. The negatively charged oxygen atom of the substrate abstracts the proton from Histidine which leads to the release of the first intermediate product. The release of the product allows water molecules to be placed in the pocket for the reaction. In the deacylation step, a water molecule helps to break the covalent bond between Serine and substrate. Histidine abstracts the proton from the water molecule, and the negatively charged oxygen of the water molecule attacks on carboxyl carbon of the substrate and forms a bond with it^13^. The attack of oxygen of water molecule on carboxyl carbon leads to the breaking acyl intermediate bond between serine and substrate which releases the product. To reset the catalytic conformation, Serine abstracts a proton from Histidine to recycle the catalytic conformation of the protein^14^.

Based on the catalytic configuration or active site architecture many engineering studies were conducted. For instance, in one of the studies reported by Santiago et al.^15^ (doi.org/10.1021/acs.biochem.8b00274) a lipase from the metagenome of Lake Arreo was designed to introduce a new engineered active site in addition to a natural active site. The double-active site enzyme was tested with the native substrate which increased the enzyme activity by twofold. The same was tested with multiple substrates and the activity was reported for many substrates.

The natural active site (NAS) of wildtype lipase enzyme (E1) (PDB Id: 5JD4) is situated in the core of the enzyme where the catalytic triad residues are Ser161, His286 and Asp256 and the oxyanion forming residues are Gly89 and Gly88. In the engineered enzyme (EE) with its natural (NAS) and newly engineered active site (EAS, PDB Id: 6I8F), the catalytic residues are Ser211, His214 and Asp25 and oxyanion forming residue is Gly207. The present study focuses on understanding the detailed mechanism of action of (EE) with the double active sites, as to what enhances the activity and its stability, by computational methods^16^. In order to explore the binding mode of the Glyceryl tributyrate (GTB) in the NAS and EAS, we employed MD simulations, atomistic conformational changes, and various computational investigations to comprehend the molecular mechanism of EE. We perform metadynamics simulations to study conformational changes and the GTB's diffusion under a bias potential. The NAS and EAS were subjected to multiple Metadynamics simulation runs under various circumstances (described in the methodology section). The pathway of substrate diffusion, where the substrate was discovered to enter NAS via EAS was examined by SMD and umbrella sampling studies. To find the reaction potentials needed for the acylation and deacylation processes, QC calculations were carried out.

## Results and discussion

### Stable binding interactions and conformational analysis of NAS and EAS residues

**Figure 2:**
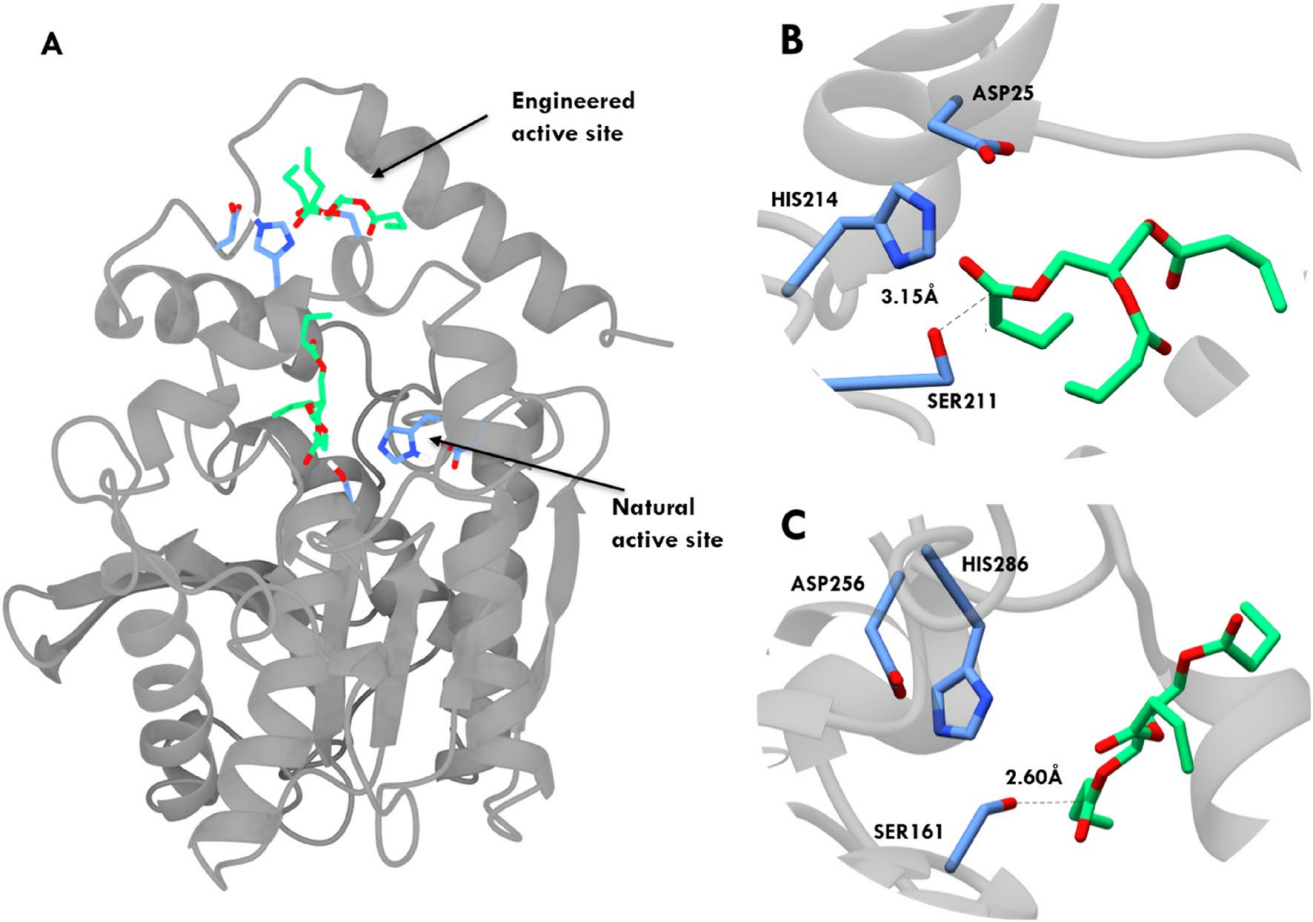
Molecular docking results of the native active site (NAS) of E1 and engineered active site (EAS) of EE. (**A**) The EAS enzyme having NAS and EAS active sites with binding conformation of GTB (**B**) EAS of EE: The active site residues (light blue sticks) in the engineered EAS of EE demonstrate interactions with the substrate molecule (green sticks), suggesting a potential catalytic binding mode. (**C**) NAS of E1: The active site residues (light blue sticks) form interactions with the substrate molecule (green sticks) in a favourable conformation, indicating potential binding and catalytic activity. The molecular docking analysis provides insights into the spatial arrangement and interactions between the active site residues and substrate molecules, highlighting the potential binding affinity and catalytic potential of both NAS in E1 and EAS in EE.

Understanding the molecular interactions between proteins and their substrates is crucial in elucidating the mechanisms of biological processes. Molecular docking was performed to investigate the binding interactions between the active site residues and the substrate in the NAS of E1 and the EAS of EE. The observed reactive distances between Ser161 and the carboxyl carbon of the substrate in NAS found to be 2.60 Å, indicating stable binding interactions (Fig. 2A,C) with the binding energy of − 3.76 kcal/mol where final intermolecular (vdW + Hbond + desolv) Energy found to be − 7.94 kcal/mol, Final Total Internal Energy is − 1.62 kcal/mol, Torsional Free Energy is + 4.18 kcal/mol and Unbound System's Energy of − 1.62 kcal/mol found for the obtained complex. Similarly, in the EAS of EE, a distance of 3.15 Å was maintained between Ser211 and the carboxyl carbon of the substrate, suggesting favourable binding configurations (Fig. 2B). The binding energy of the complex found to be − 1.72 kcal/mol consists of final intermolecular (vdW + Hbond + desolv) energy of − 5.90 kcal/mol, Final Total Internal Energy is − 1.84 kcal/mol, Torsional Free Energy is + 4.18 kcal/mol and Unbound System's Energy of − 1.84 kcal/mol. To validate the reliability of the docking results, MD simulations and other calculations were conducted. It was found that the maintained distances were in agreement with the conformations observed during the MD simulations. The consistency between the docking results and the MD simulations further supports the stability and reliability of the predicted binding interactions. The specific distances and conformations obtained from molecular docking provide insights into the key interactions between the active site residues and the substrate. These interactions are crucial for understanding the binding mechanism and substrate specificity of the protein. The stability of these interactions is essential for efficient enzymatic catalysis and substrate recognition.

### Structural stability and reactive distances in molecular dynamics simulations

**Figure 3:**
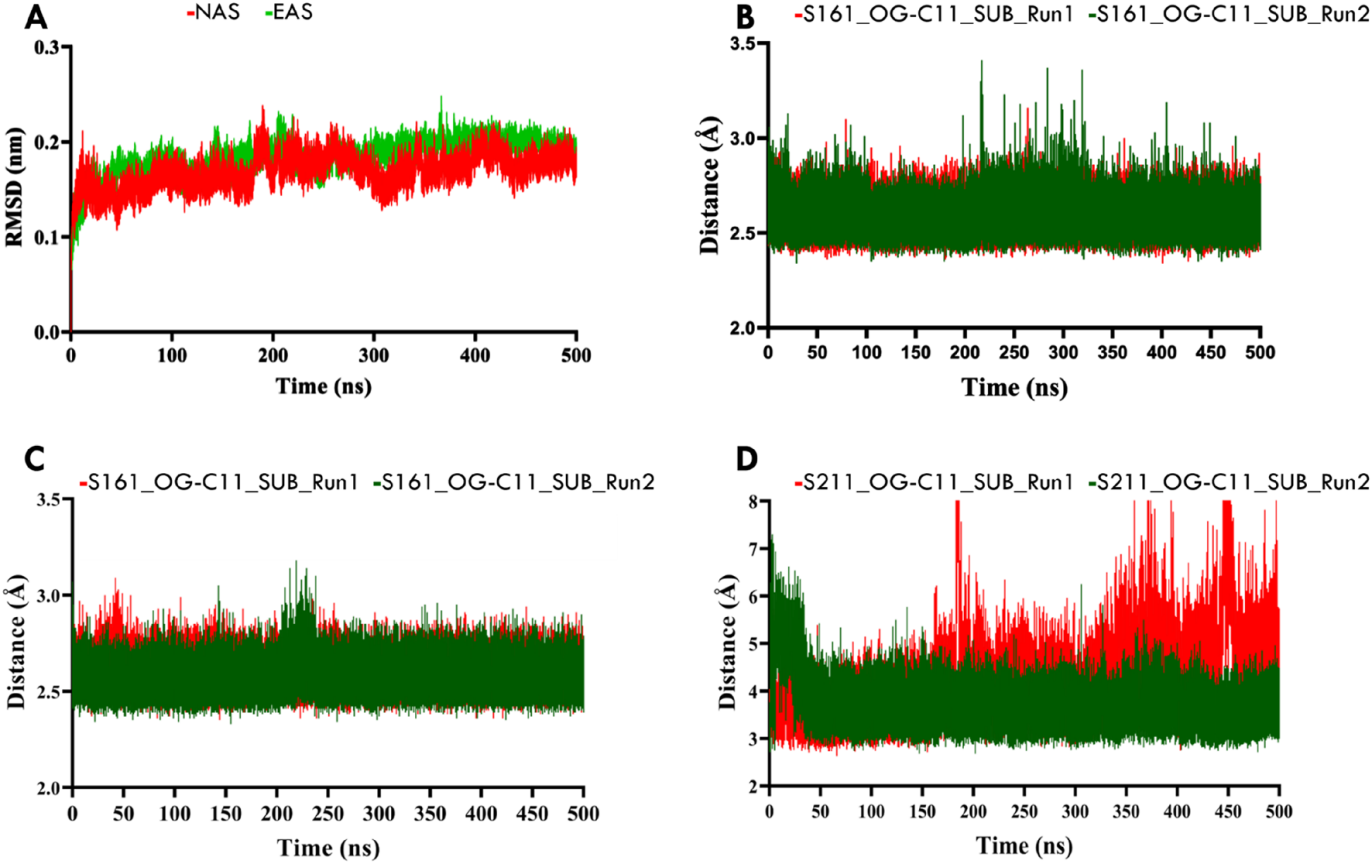
The distance and RMSD calculated for the MD simulation of 500 ns*2 on E1 and EE complexes. (**A**) The RMSD of the NAS and EAS simulation shows the consistency of the MD simulations where the first frame of the simulation is used as a reference to calculate the RMSD across the simulation time in both cases. (**B**) the distance between catalytic residue Ser116-OG with Carboxyl carbon of GTB in NAS of E1, (**C**) the distance between catalytic residue Ser116-OG with Carboxyl carbon of GTB in NAS of EE and (**D**) the distance between catalytic residue Ser211-OG with Carboxyl carbon of GTB in EAS of EE. The average RMSD of 0.2 nm shows the stability of the E-S Complex across the simulation time where distance is also maintained as supportive data. The dynamics of the substrate in the EAS of EE were found to be not as stable as NAS in the simulation.

To assess the structural stability of E1 and EE throughout the simulations, the root-mean-square deviation (RMSD) of the Cα atoms was calculated by considering the first frame of the MD simulation as a reference structure. The overall RMSD analysis indicated that the protein conformations remained stable during the simulations, with values below 0.2 nm for both E1 and EE (Fig. 3A). The low RMSD values suggest that the protein structures maintained their overall conformation throughout the simulation, validating the subsequent analyses. The evaluation of structural stability using RMSD calculations revealed that both NAS of E1 and NAS of EE exhibited stable conformations. The reactive distance between the carboxyl carbon of the substrate and the reactive oxygen of Ser161 in NAS of E1 was consistently maintained at an average distance of 2.587 ± 0.73 Å (Fig. 3B). Similarly, in NAS of EE, the reactive distance between the carboxyl carbon of the substrate and Ser161 was maintained at an average distance of 2.650 ± 0.62 Å (Fig. 3C). These stable reactive distances indicate high stability of the substrate within the NAS region. In the EAS of EE, the distance between the carboxyl carbon of the substrate and Ser211 was measured. The average distance was found to be 3.821 ± 0.58 Å, suggesting a relatively stable interaction between the substrate and Ser211 in the EAS region. The maintenance of reactive distances between the substrate and the catalytic residues Ser161 and Ser211 in NAS and EAS, respectively, supports the stability of the substrate in these regions. The observed stability in NAS is evident in the substrate's conformation, as depicted in Figure 3B. However, in one of the simulations, the substrate was observed to move outside the active site after 125 ns of simulation (Fig. 3D) in the EAS of EE. This observation suggests potential dynamic behaviour and weaker interactions in this region, possibly explaining the lower stability of the substrate compared to NAS. The dynamic movement of the substrate outside the active site in EAS indicates that the EAS region may not provide the same level of stabilization and favourable binding conformation as observed in NAS. This dynamic behaviour could potentially affect the catalytic efficiency of the enzyme. The molecular dynamics simulations demonstrated the overall structural stability of both E1 and EE. The maintenance of specific reactive distances in NAS and EAS supported the stability of the substrate in these regions. However, the observation of the substrate moving outside the active site in EAS of EE suggests a potential dynamic behaviour and weaker interactions, which may have implications for the catalytic efficiency of the EAS active site of the enzyme.

### Steered MD and umbrella sampling studies demonstrate seamless diffusion of the substrate to the E1 via EE

**Figure 4:**
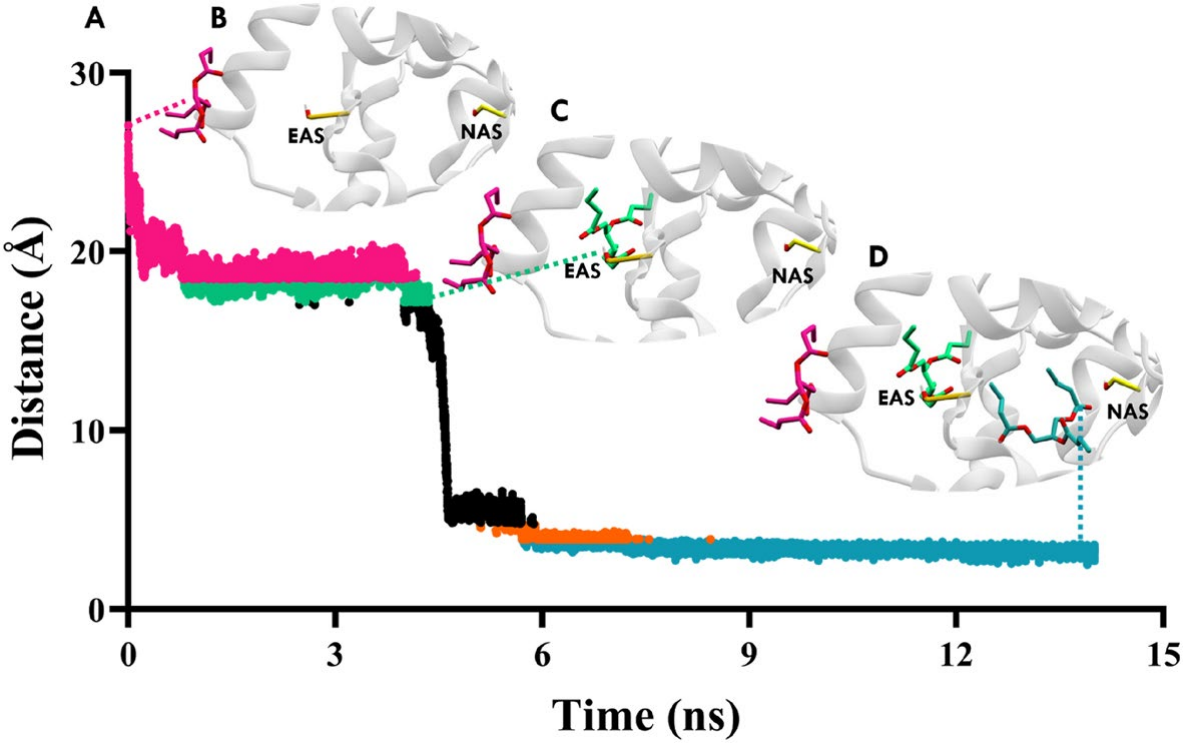
Distance analysis from SMD simulations illustrating the substrate molecule's movement from non-interacting region towards NAS. (**A**) Distance between the carboxyl carbon of the substrate and the reactive oxygen of Ser161 (catalytic residue of NAS) throughout the simulation. Different colours represent distinct conformations of the substrate in various regions: magenta (**B**) denotes the substrate outside EAS, green (**C**) represents the substrate in a near attack conformation in EAS, including translocation from EAS to NAS (black), orange signifies the substrate close to NAS, and steel blue (**D**) indicates the substrate in a near attack conformation in NAS. The substrate (magenta sticks) exhibits movement from outside the protein towards forming a near-attack conformation in EAS (substrate in green sticks, Ser211 in yellow sticks) and subsequently transitioning into a near-attack conformation in NAS (substrate in steel blue, Ser161 in yellow sticks). This analysis provides insight into the dynamic behaviour of the substrate within the protein, highlighting its conformational changes and interactions with key catalytic residues in both EAS and NAS.

**Figure 5:**
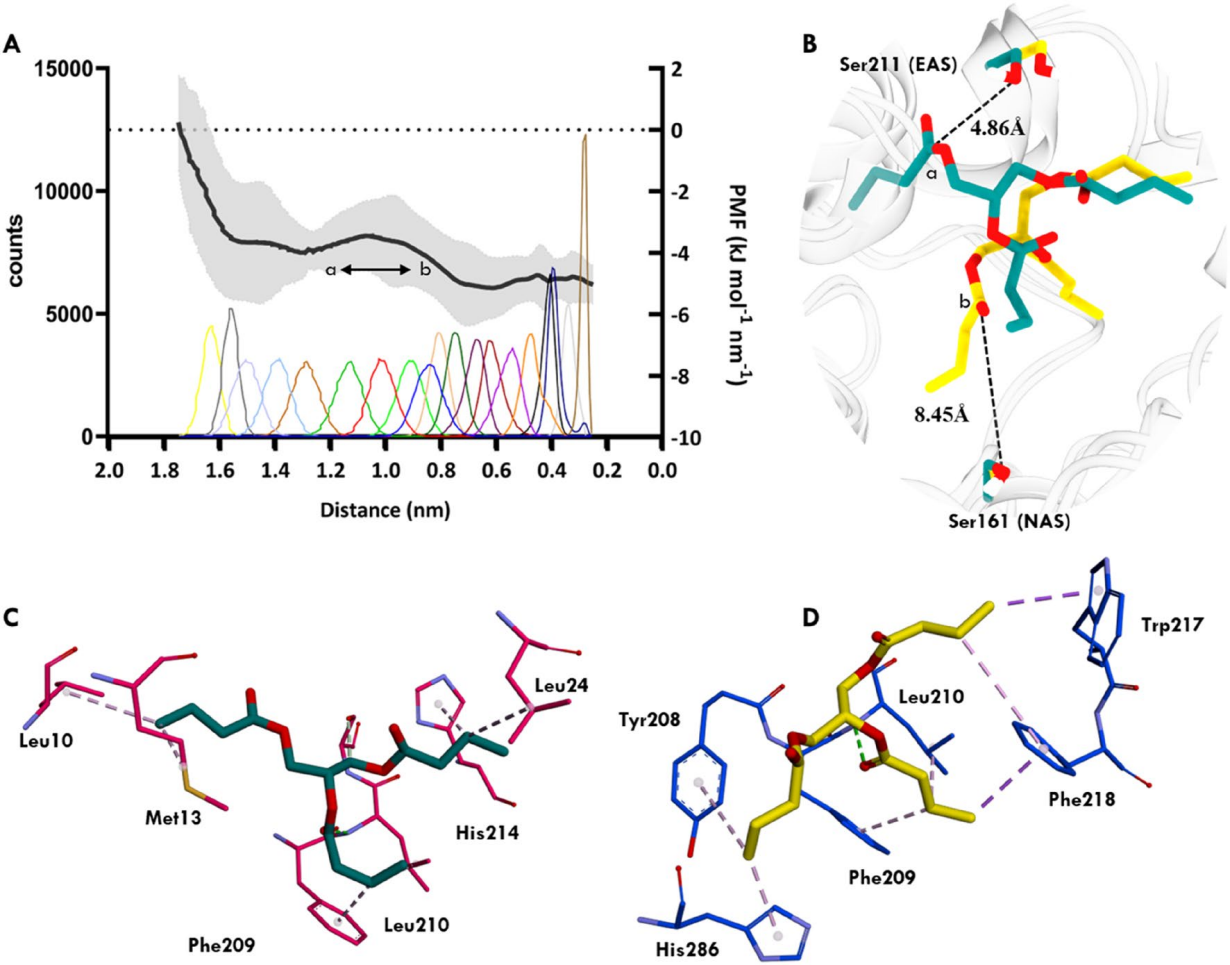
The translocation of the substrate from the EAS to NAS (~ 17 Å) studies using umbrella sampling where the high energy regions could be the scope of regions for engineering purposes. (**A**) The PMF graph overlaid above the histogram to understand the energy associated with the movement of the substrate. The PMF profile shows the energy taken by the substrate across the path and the histogram shows the count of frames within the specific window. To ensure statistical significance, the PMF values are derived from the mean of three stimulations. An arrow has been used to highlight a negligible barrier or negligible increase in the energy at the 1.2 nm to 0.8 nm region (highlighted as "a and b"). (**B**) The conformation of “a and b” were extracted, revealing the GTB's conformation in two distinct regions: "b" is 8.45 Å from the NAS and "a" is close to the EAS active site at 4.8 Å. C and D display interacting residues of "a and b". The residues surrounding the substrate in both the conformation highlight strong hydrophobic interactions between the substrate and the residues in the tunnel.

To investigate the movement of the substrate from EAS to NAS, a combination of steered molecular dynamics (SMD) and umbrella sampling techniques was employed. Initially, the substrate was positioned away from the active site, approximately 27 Å away from NAS of EE where it is not interacting with any amino acids, to study the diffusion of the substrate from the solvent into the protein (Fig. 4A,B). The substrate was allowed to freely move in the x, y, and z directions without any constraints. Multiple runs were performed to facilitate substrate movement towards NAS of EE. During the simulations, the substrate gradually adopted a near-attack conformation in EAS (Fig. 4C) and translocated towards NAS (Fig. 4D). After 14 ns of simulation, the substrate was observed to form a near-attack conformation in NAS. To further understand the behaviour of substrate across the path, the trajectory was discreted and subjected to Umbrella Sampling. Umbrella sampling studies have provided insights into the residues along the translocation pathway that are responsible for substrate movement and the energy required for the substrate to adopt a near-attack conformation. Specifically, umbrella sampling was conducted to investigate the translocation of the GTB from EAS to NAS, covering a distance of approximately 17 Å (Fig. 5A). Conformations were sampled at intervals of 0.5 Å along the distance between Ser161 and the carboxyl carbon of the substrate which brings the overall number of windows 34. The successful diffusion of the substrate to the NAS through the EAS, as demonstrated by the steered MD and umbrella sampling studies, elucidates a plausible translocation pathway. The initial placement of the substrate away from the active site and its subsequent movement towards the NAS highlights the directionality of substrate binding and the role of EAS as an entry point. The umbrella sampling studies provide valuable information about the energy landscape and the specific residues involved in the translocation process, further confirming the accessibility and stability of the translocation pathway.

The Potential Mean Force (PMF) is the energy profile that the substrate requires to translocate from EAS to NAS. The energy expended on the translocation path from EAS to NAS is displayed by the PMF. As the substrate leaves the EAS active site and enters the tunnel to reach NAS, it is found that the area between 1.2 and 0.8 nm is critical. A small negligible increase in energy might be seen since the GTB found strong interactions there, which is close to the EAS (Fig. 5B). Leu10, Met13, Leu24, Phe209, Leu210, and His214 are the interacting residues for the conformation found in the 1.2 nm region (Fig. 5C). In contrast, Tyr208, Phe209, Leu210, Trp217, Phe218 and His286 are found in the 0.8 nm region (Fig. 5D). A network of hydrophobic interactions between several different sets of residues and the substrate is formed in both the cases, these interactions are important. Also, these residues can be engineered further for the smooth entrance of the substrate.

### Energetics of reactions in NAS and EAS: insights from QM/MM simulations

In the exploration of molecular reactions using simulations, the QM/MM method, a fusion of quantum mechanics (QM) and molecular mechanics (MM), provides an insightful perspective. Our primary aim with this study was to decipher the energetic nuances of reactions occurring within NAS and EAS.

**Figure 6:**
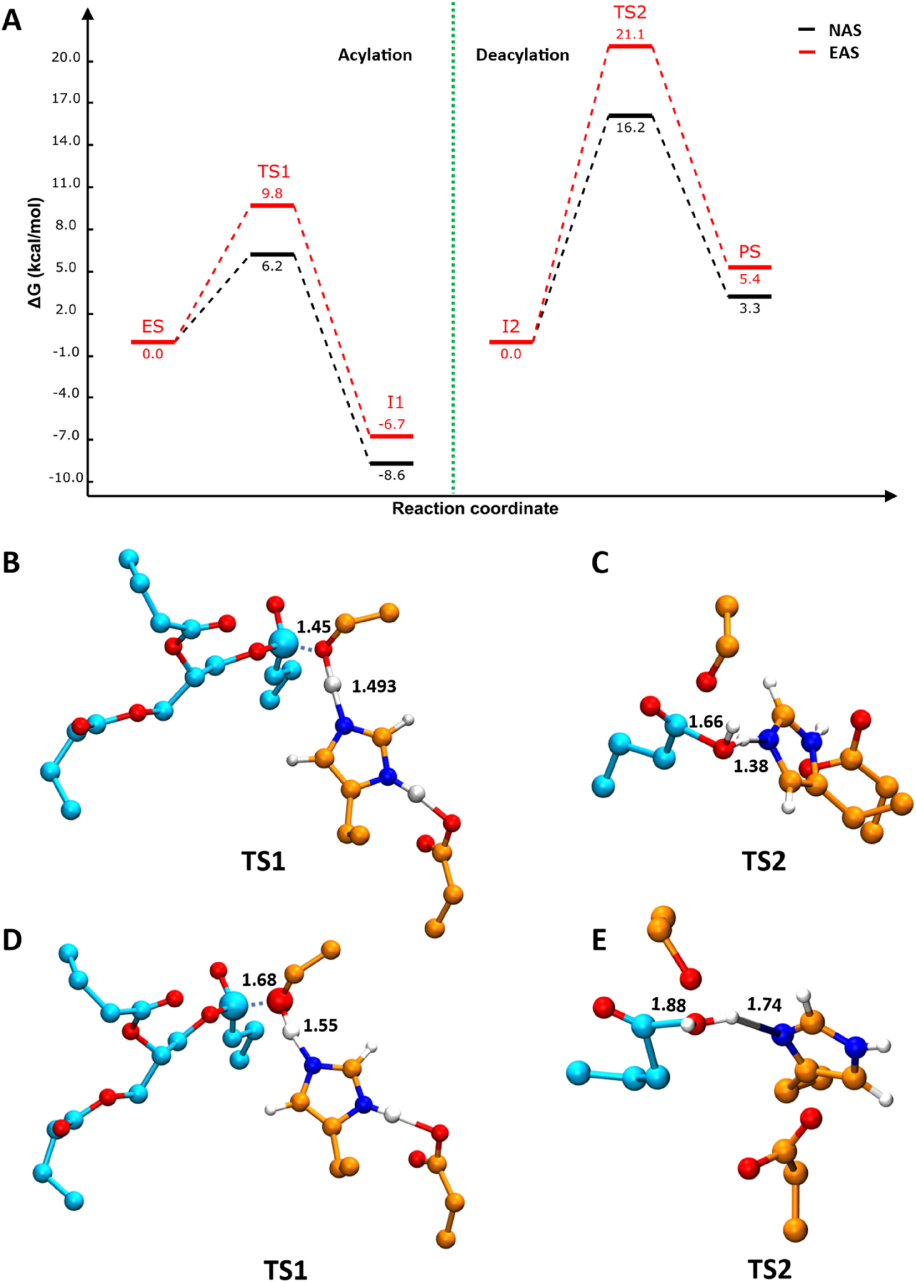
(**A**) Reaction free energy profile for acylation and diacylation mechanism of lipase reaction obtained from QM/MM simulation, (**B** and **C**) The transition state 1 and 2 conformations from NAS, D and E) Transition state 1 and 2 from EAS. QM/MM simulations conducted on both NAS and EAS, the free energy level of intermediate2 (I2) is set to zero to highlight the barrier for deacylation reaction. NAS with strong binding conformation in the pocket was found with minimal energy as compared to EAS. EAS was found with higher energy for all the transition states, intermediate states and the product state (PS) across the acylation and deacylation pathway.

Upon conducting QM/MM simulations, we observed that the NAS active site conformation is equilibrated for 3000 steps, reflected by lower QM/MM energy values (Fig. 6A). This energetic stability contrasts sharply with the EAS, where the conformation is external. Due to fewer interactions available to stabilise the substrate in EAS, there is a pronounced increase in energy levels, as demonstrated in our QM/MM data.

The reaction scheme in Fig. 1 illustrates the ligand-bound and unbound states. The bound state (ES complex) is the state in which the substrate is brought into the active site, as demonstrated in Fig. 2 (near attack conformation from molecular docking studies) for both NAS and EAS. The first intermediate product, which forms the acyl-enzyme intermediate, is released as a result of nucleophilic attack by the catalytic residue Ser on the substrate and deprotonation of His by the negatively charged oxygen atom of the substrate (TS1 Fig. 6B, D).

The leaving group from acylation (I1-acyl-enzyme intermediate) has been removed and H_2_O is modelled in the active site to obtain (I2). The H_2_O molecule helps to break the covalent bond between Ser and substrate. His abstracts the proton from the water molecule, and the negatively charged oxygen of the H_2_O attacks the carboxyl carbon of the substrate and forms a bond with it which is the TS2 (Fig. 6C, E). The attack of oxygen of the H_2_O on carboxyl carbon leads to the breaking acyl intermediate bond between Ser and substrate forming a Product state (PS) which is the unbound state.

Furthermore, we discerned a pattern in energy demands for the reaction steps in both NAS and EAS. While acylation is more energetically favourable, demanding lesser energy, the deacylation step is notably more energy-intensive. QM/MM simulations have unveiled the higher energy requisites for reactions in EAS, especially in the acylation and diacylation steps, highlighting the critical role of substrate stabilization and active site conformation in influencing reaction energetics.

### Metadynamics simulations revealing conserved binding interactions and substrate stability in the active site of E1 and EE

**Figure 7:**
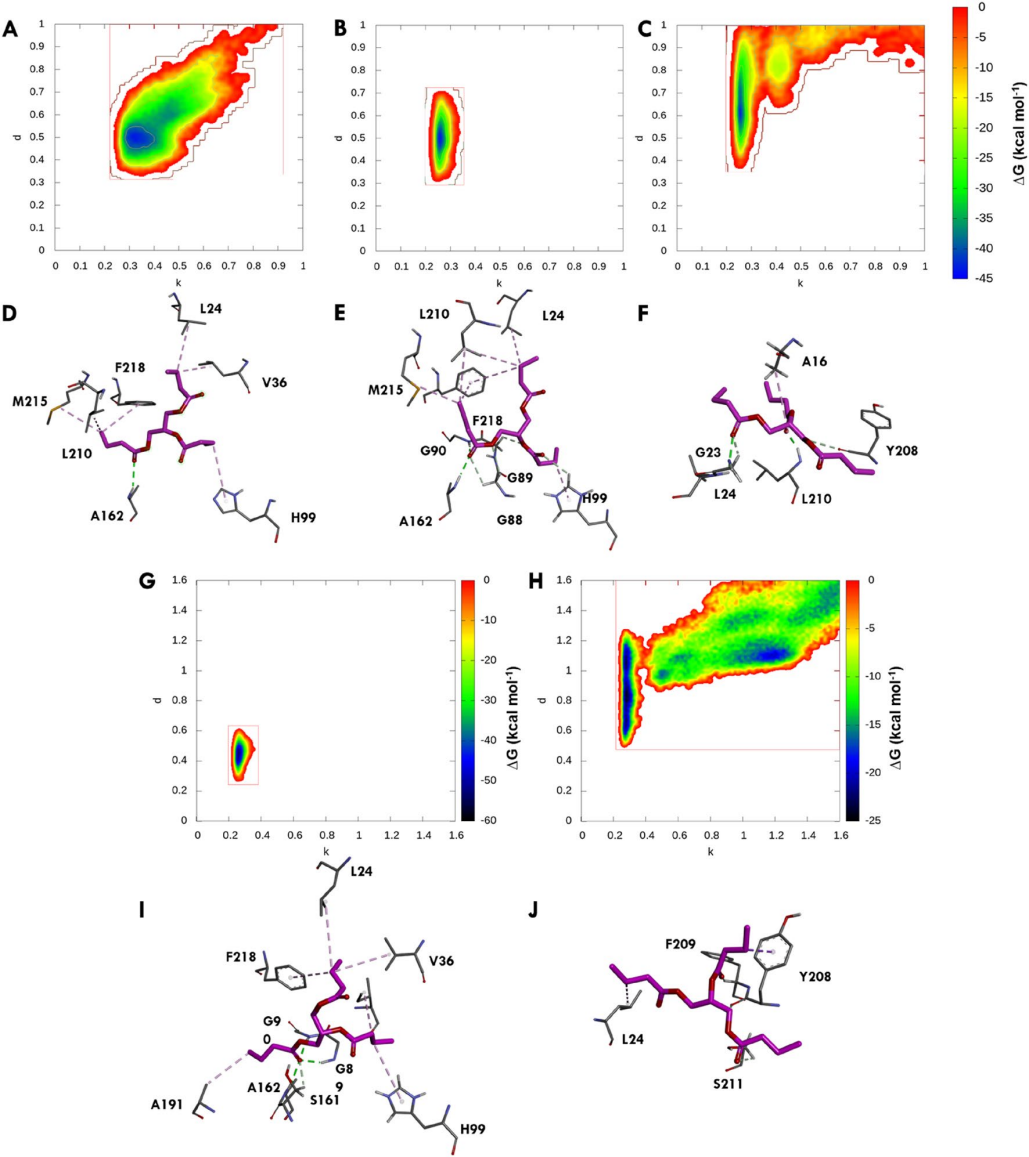
Metadynamics analysis of the native active site (NAS) in E1 and engineered active site (EAS) in EE. (**A**) Metadynamics studies on NAS of E1in S1 simulation reveal multiple minima, representing stable substrate conformations within the pocket. The lowest free energy minima indicates a stable conformation. (**B**) Metadynamics analysis of NAS of EE in simulation S2 shows a similar distribution of local minima, with the lowest free energy suggesting a stable conformation comparable to E1. (**C**) In S3 simulation, the substrate in EE's EAS exhibits dynamic behaviour, moving outside the pocket, indicating potential instability compared to NAS. (**D**) Interactive residues in the NAS of E1 includes Leu24, Val36, Gly88, Gly89, Gly90, Ile93, His99, Ser161, Ala162, Ala191, Phe218, His286, Gly287, Ser290, and Leu291. (**E**) Interactive residues in the NAS of EE are similar to E1, suggesting comparable substrate binding interactions. (**F**) In S3 simulation, the EAS of EE shows fewer interactive residues may contribute to increased substrate dynamics observed in metadynamics, the interactive residues found are Ala16, Gly23, Leu24, Tyr208, Phe209, and Leu210. (**G**) In the S4 simulation, NAS of EE behaves similarly to S1 and S2 simulations having the lowest free energy minima. (**H**) In the same simulation EAS moves outside the active site. (**I**) The Substrate in NAS was highly stable by interacting with a greater number of residues and forming a stable conformation, (**J**) The EAS shows fewer interactions which makes it more dynamic and unstable in the active site. CV1 measures the distance between the COM of active site residues and the substrate (denoted as k), while CV2 measures the COM distance between the catalytic residue (Ser-O) and substrate carboxyl carbon (denoted as d). These variables characterize substrate conformational states and their free energies.

Metadynamics simulations were performed on NAS in S1 simulation to explore the local minima of the substrate within the pocket. The binding conformations of the substrate in NAS of E1 and EE were found to be similar in S1 and S2 simulations. The collective variables (CVs) used in the metadynamics simulations are defined as follows: CV1, which represented the distance between the centre of mass (COM) of the backbone atoms of the active site residues and oxyanion hole forming residues, and the COM of the substrate. CV2 represented the COM distance between a reactive atom of the catalytic residue (Ser-O) and the carboxyl carbon of the GTB. The defined CVs allowed for the identification of local minima where the GTB could stay longer and form a higher number of interactions. The proximity of the local minima to the origin (lower-left corner) of the graph indicated better maintenance of the near-attack conformation. In the NAS of E1 in S2 simulation, the local minima were found to be the least distant from the origin, with the lowest free energy observed was − 33.80 kcal mol^−1^ (Fig. 7A) within 4 Å of both the CVs. Similarly, NAS of EE in the S2 simulation exhibited a lower free energy of − 43.319 kcal mol^−1^ (Fig. 7B) within 4 Å of both the CVs. The energy profile for EAS of EE displayed a similar free energy of -45.364 kcal mol^−1^ (Fig. 7C) within 5 Å of both the CVs. According to the energies above, the substrate maintained a conformation with a higher number of contacts within 4Å of defined CVs (Fig. 7D,E); in contrast, EAS was discovered at 5 Å, indicating that the substrate was migrating away and was not as stable as it was in NAS (Fig. 7F). In S1, S2 and S3 simulations CVs and bias potential were applied on single active sites and their respective substrate which gave independent insights into the respective dynamic behaviour of the substrate. The S4 simulation was conducted to study the probability of substrate stability when both the active sites were subjected to respective CVs and bias potentials. In the S4 simulation, the dynamic behaviour of the substrate in NAS remains the same as depicted in S1 and S2 simulations (Fig. 7G). However, the substrate in EAS moved away from the active site and did not attain local minima in the active site (Fig. 7H). The lowest free energy observed within 4 Å of CV1 and CV2 of NAS with free energy of -50.071 kcal mol^−1^ and in EAS, free energy of − 0.960 kcal mol^−1^ was found at 5 Å of both the CVs. This also concludes that the GTB in EAS was not as stable as in NAS. The GTB conformation in NAS of E1 and EE showed minimal movement indicating highly stable complex and stable positioning within the active site (Fig. 7I). However, in EAS of EE, the substrate was observed to move towards the outside of the active site, as it was interacting with fewer amino acids and more dynamic water molecules (Fig. 7J). The binding conformation of the substrate in NAS of E1 and EE was similar, indicating conserved binding interactions. The comparison of energy profiles and minima between NAS of E1 and EE suggested that the substrate achieved a lower energy conformation and greater stability in the NAS pocket. The increased substrate dynamics and movement outside the pocket observed in EAS of EE could be attributed to the presence of fewer interacting residues, limiting substrate stabilization and potentially hindering the reaction. Simulations conducted on NAS provided insights into the local minima and binding conformations of the substrate. The similarity in binding conformations between E1 and EE suggested that the designed EAS in EE provided the same level of stability as NAS, potentially favouring the substrate's ability to adopt a low-energy conformation and facilitate the reaction.

### Characterization of a translocation pathway from NAS to EAS: insights from tunnel studies

**Figure 8:**
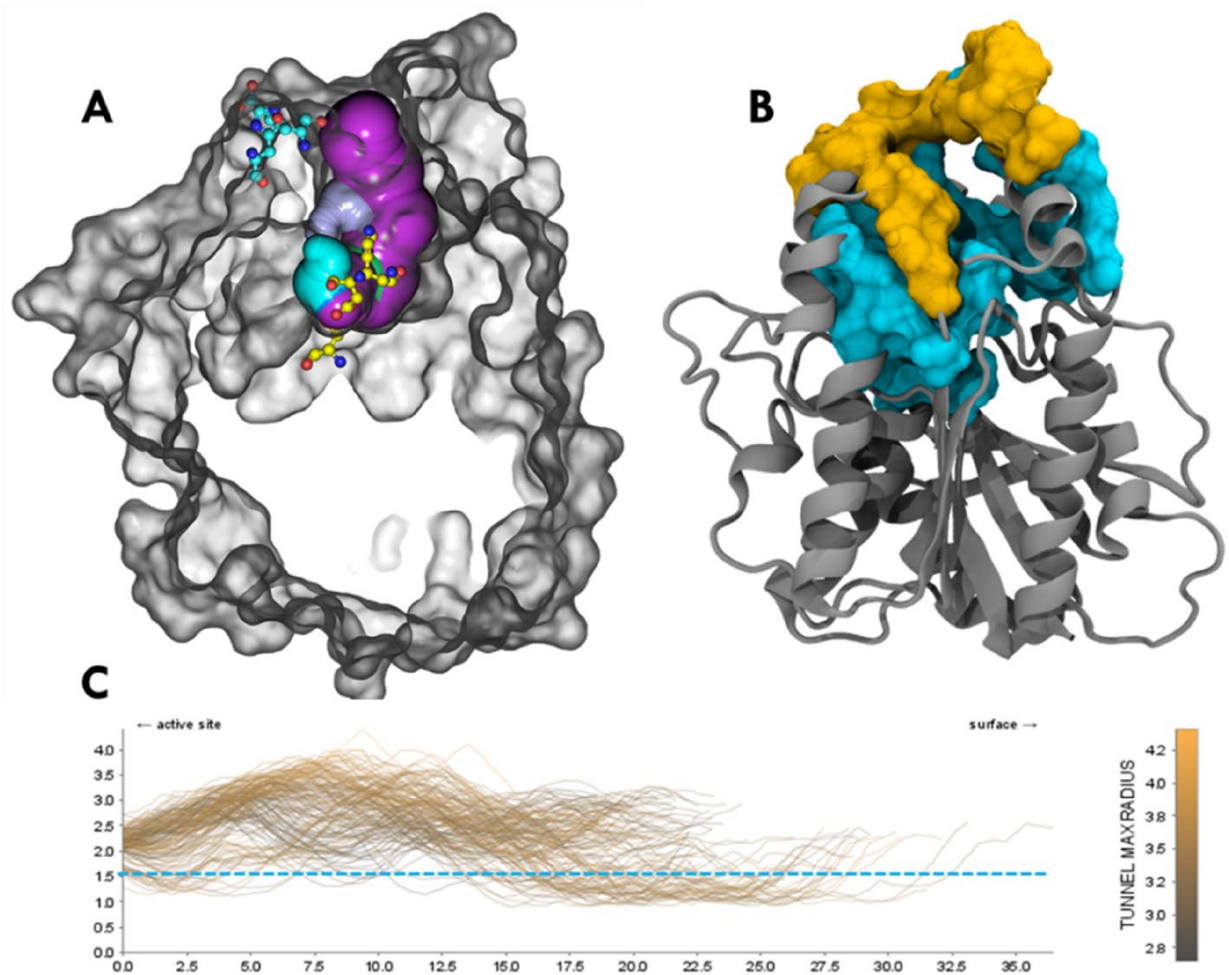
Channels and tunnels in the protein structure. The figure illustrates the channels and tunnels identified in the protein structure during the simulation. (**A**) The active site residues of the native active site (NAS) are shown in yellow sticks, while the engineered active site (EAS) is represented by cyan sticks. The purple-coloured tunnel indicates the pathway connecting the NAS and EAS. The protein structure is depicted in a clipped form for clear visualization of the tunnel. (**B**) Important tunnel entry regions of the protein (shown in yellow) and tunnel (shown in dark cyan) are highlighted on the protein with surface representation. These residues play crucial roles in regulating substrate passage and creating the structural pathway. (**C**) Resents the radius of the tunnel throughout the simulation time. The blue dotted line represents the average radius of tunnel curvature, which measures 1.528 Å. This analysis provides insights into the stability and geometry of the tunnel during the simulation.

Tunnel studies were conducted to investigate and validate the presence of a translocation pathway for the substrate from NAS to EAS. Structurally, EAS was identified as being located near the substrate entry channel on the enzyme's surface (Fig. 8A), while the tunnel connecting NAS to the outside region, terminating near EAS, remained stable throughout the simulation. The residues involved in tunnel formation included Leu12, Ala16, Arg21, Leu24, Val36, Met39, Ser40, Gly89, Phe218, Ala253, Asp256, Pro257, Leu258, Ile285, His286, Gly287, Phe294, while the tunnel entry forming residues comprised Asp11, Asp14, Thr17, Arg18, Gly20, Pro22, Gly23, Asp25, Thr26, Tyr208, Ser211, Ala213, His214, and Tyr255 (Fig. 8B). The analysis of tunnel properties revealed an average bottleneck radius of 1.4730 Å, an average tunnel length of 25.75 Å, and a consistent tunnel curvature of 1.528 Å throughout the simulation (Fig. 8C). The identification and characterization of these tunnels and channels in the protein structure provided strong evidence for the existence of a translocation pathway from NAS to EAS. The presence of tunnel-forming residues and tunnel entry forming residues shed light on the key amino acids involved in facilitating the substrate translocation. The characterization of tunnel properties, including radius, length, and curvature, emphasized the feasibility of substrate movement through the tunnel and its potential impact on the overall enzymatic activity.

## Discussions

The presence of a double active site in the engineered variant EE provides a unique feature compared to the native enzyme E1. The introduction of the engineered active site (EAS) offers an additional binding pocket, potentially allowing for simultaneous binding and catalysis of multiple substrates or alternative reaction pathways. This feature opens up new possibilities for enzyme engineering and expands the scope of enzymatic reactions that can be catalysed. In lipases, the active site is typically located deep within the core, and substrate flow is regulated by a functional lid. The presence of a lid in an open conformation allows substrate entry. In the absence of significant lid movement, additional substrates should remain outside the active site. This discussion provides context for the structural arrangement of NAS and EAS and emphasizes the importance of stable substrate conformations for efficient catalysis. The designed EAS is not a pocket like the NAS, which makes the substrate dynamic and prevents it from adopting the lower-energy conformation. The absence of significant secondary structure and pocket-forming residues may limit the stability of the substrate binding conformation. Consequently, the substrate molecule exhibits greater dynamics and mobility in EAS, hindering the attainment of the most stable conformation required for efficient catalysis. This observation is consistent with the reduced number of interacting residues in EAS compared to NAS. Analysis of the interacting residues in NAS and EAS revealed distinct sets of amino acids involved in substrate binding. NAS exhibits a higher number of interacting residues, suggesting greater stability and a lower energy conformation for the substrate compared to EAS. This observation further supports the hypothesis that NAS is the preferred binding site for efficient catalysis. The presence of fewer interacting residues in EAS may limit substrate stabilization and affect catalytic efficiency. The metadynamics simulations provided insights into the stability of the substrate in NAS and EAS. The substrate molecule explored different minima within the pocket due to the bias factor introduced in the simulations. While the substrate was occasionally observed to move outside into the bulk water in EAS, it remained considerably more stable in the NAS pocket of both E1 and EE. These findings suggest that the NAS pocket provides a more favourable environment for substrate stability, promoting the attainment of a low-energy conformation required for efficient catalysis. The SMD experiments demonstrated the route through which the substrate accesses the active site. The substrate primarily entered through EAS and entered into the NAS. The caver tunnel analysis further supported the presence of EAS on the substrate's entry channel. However, the substrate's ability to assume a viable energy configuration for the reaction to proceed was found to be rare, consistent with wet lab investigations that showed only a modest increase in activity (8–15%). The presence of a double active site raises questions about substrate preference and selectivity. Are both active sites capable of accommodating the same substrate, or do they exhibit different substrate preferences? Further investigations into the substrate specificity of the double active site could provide valuable insights into the potential applications of the engineered variant. The potential synergistic effects between the native and engineered active sites in EE are also worth exploring. The presence of multiple active sites within the same enzyme molecule can facilitate cooperative interactions and enhance the overall catalytic efficiency. Investigating potential synergistic effects between the native and engineered active sites could lead to the discovery of novel catalytic mechanisms and improved enzymatic activity. Moreover, evaluating the impact of the double-active site on the overall catalytic rate and efficiency of the enzyme is crucial. Comparative studies between E1 and EE, including kinetic measurements and rate analysis, would provide insights into the influence of the double-active site on enzymatic activity. The successful creation of the double active site in EE demonstrates the potential of rational enzyme design and engineering strategies. Understanding the structural and functional aspects of the engineered active site can guide future enzyme engineering efforts, enabling the development of customized enzymes for specific applications. The knowledge gained from the characterization of the double active site in EE can be utilized in the design of novel biocatalysts with enhanced capabilities.

## Conclusion

The investigation of the double active site in EE has provided valuable insights into its structural arrangement, substrate specificity, and potential catalytic effects. The presence of a double-active site offers new opportunities for expanding the range of catalysed reactions and designing tailored enzymes for specific applications. Further studies are warranted to explore the substrate preferences, catalytic efficiencies, and synergistic effects associated with the double-active site to fully harness its potential in biocatalysis.

## Methods

The crystal structure of E1 and EE (PDB Id: 5JD4 and 6I8F respectively) was collected from the PDB database. Glyceryl tributyrate (GTB) was used as the substrate wherein E1 and EE showed Kcat of 440.83 ± 2.3 and 465.31 ± 6.77 respectively. EE improved the activity by 15% (Santiago et al.^15^, Supplementary Table 01). Molecular Docking studies were performed to attain near-attack conformation for both E1 and EE. The least energy conformation was considered for Molecular Dynamics (MD) studies, steered MD simulations, umbrella sampling and Metadynamics. MD simulations showed the stable and closest interactions of the substrate with the active site residues, Steered Molecular Dynamics (SMD) with Umbrella Sampling (US) was used to study the association path of the substrate, and Metadynamics revealed different regions on the protein where the substrate adopts a least energy conformation and Tunnel analysis revealed all the possible paths that the substrate could have travelled.

### Molecular docking studies

Molecular docking studies were carried out using Autodock4 on E1 with NAS, where the catalytic residues are Ser 161, His 286 and Asp256, and with EE possessing EAS, where the catalytic residues are Ser 211, His 214 and Asp25^17,18^. The substrate in both instances was GTB, which was sketched using the Marvin tool and optimised using the Avogadro tool^19^. For E1 grid box was defined as 102, 70, 78 in XYZ direction with the grid spacing of 0.225 Å and the grid centre was maintained at 63.419, 79.827, 8.194. For EE the grid box was defined as 38, 42, 40 in XYZ direction with a spacing of 0.375 Å and grid centre 1.914, 15.491, and − 15.872 was maintained^20,21^.

### Molecular dynamics simulations

The Molecular Dynamics (MD) simulations were conducted using GROMACS 2019.4^22–25^. The simulation was performed using AMBER99sb force fields^22,26^ with the water model TIP3P^27–29^ to mimic the natural environment. The GAMESS tool and antechamber 1.24 were used for generating parameters for the GTB molecule and then converted into GROMCAS format^30–34^. TIP3P water model with a box volume of 1000 nm^3^ was used to solvate the ES-Complex. In the case of E1 and EE, the system was neutralized using a 0.15 mM concentration of NaCl ions. 3593 water molecules, 27 Na^+^ ions and 15 Cl^−^ ions were added in the E1 system along with the enzyme-substrate complex which makes a total of 15597 atoms and in the case of the EE system 3935 water molecules, 32 Na^+^ and 17 Cl^−^ ions were added along with enzyme-substrate complex which brings the total number of atoms to 16632. Energy minimization of the system was performed using the steepest descent algorithm with the convergence energy cut-off of 1000 Kj mol^−1^ within 1000 steps^35^. Equilibration was done using NVT and NPT (N = constant number, V = constant volume, T = constant temperature, P = constant pressure)^36^. The NVT and NPT equilibration steps were performed for 30 ns at 300 k^37^. The short-range cut-off values of van der Waals were set to 1.0 and columbic interactions were calculated using the PME (Particle Mesh Ewald) algorithm^38,39^. LINCS algorithm was used for all bond length constraints and 2fs was set for each time step of simulations. MD simulations of the system were conducted for 500 ns X 3 runs at 300K. The conformations were saved for every 2ps. GROMACS default analysis commands, UCSF Chimera^40–42^, and VMD tools were used for the analysis^43,44^. The MD analysis such as Principal component analysis, distances^45–48^, and pocket analysis using Caver visualizer conducted to understand the structural behaviour of the ES-Complex^49–51^.

### QM/MM simulation using CPMD

The QM/MM simulations were conducted using the CPMD tool, DFT-based AIMD implementing the Car-Parrinello scheme with metadynamics was performed using the CPMD 4.3.0 program. The system includes the three catalytic amino acids and the substrate. The alpha-carbons were turned into methyl groups and were fixed in space considering only the side chains of amino acids. To calculate the electronic ground state and optimise the electronic density of the initial structure, wavefunction optimisation was done. The optimised structure (when the energy converged under a value of 3.0 × 10^−6^a.u.) was equilibrated scaling the temperature to 300K. The simulations were conducted under periodic boundary conditions in a canonical ensemble (NVT). The simulation box was an orthorhombic symmetry (10 Å to 15 Å a side). Becke, Lee, Yang, and Parr (BLYP) gradient-corrected functional was used for the exchange and correlation terms with a density cutoff of 5.0 × 10^−6^ along with Vanderbilt ultra-soft pseudopotential and plane wave basis set with a kinetic energy cutoff of 25 Rydberg was employed. The temperature was controlled using a Nose-Hoover thermostat, it was set at 300 K for the ions and for electrons target fictitious kinetic energy of 0.06a.u was maintained. For the metadynamics simulations, history-dependent potentials were added at every 20 MTD step with Gaussian hills height 0.01 kcal/mol and width 0.2 atom units. Free energy surface in collective variable space was reconstructed using the Vreco CPMD program by Dr N. Nair, which is provided by CPMD^52^.

### Metadynamics

Metadynamics simulations were performed to understand the free energy landscape of the active site using PLUMED 2.7^53–55^. In traditional molecular dynamic simulations, the dynamics of the substrate tend to attain a singular local minima. Exploration of different conformations of the substrate in the active site is possible when a bias force is applied to it^56^. Metadynamics was conducted on E1 and EE where 2 different collective variables (CVs) were configured to understand the free energy landscape. For E1 and EE, CV1 is the distance between the centre of mass (COM) of backbone atoms of active site residues along with oxyanion hole forming residues and COM of the substrate. CV2 is the COM distance between the reactive atom of catalytic residue Ser specifically Ser-O and the carboxyl carbon of the substrate. The bias factor of 2.0 and temperature of 310 k with the minimum Gaussian bin height of 1 Å were maintained as starting parameters^57,58^. Metadynamics simulations were used to study the dynamics behaviour of substrate GTB in E1 and EE. Simulations were conducted on multiple conditions as explained below in Table 1 to study the dynamical behaviour of GTB when it is present in different active sites under given bias potentials^59,60^.

**Table 1:** The metadynamics simulations were conducted on E1 and EE with different conditions where CVs were applied on NAS and EAS. The E1 system contains only NAS which is a simulation in S1 simulation where CV is applied on GTB and Ser161, His256, Asp284, Gly88, Gly89 and Gly90. S2 simulation contains an EE complex where both the active site holds substrate but focused only on NAS of EE by applying the same CVs as S1 simulation. In the S3 simulation, CVs were applied on EAS where CVs were applied on GTB of EE and Ser211, His214, Asp25, Phe209, Tyr208, and Gly207. In S4 simulations, CVs applied on NAS and EAS both systems parallelly where CVs on NAS were taken as S1 simulation and CVs on EAS taken as S3 simulation. For the above S1, S2, S3 and S4 simulations two CVs were applied as explained in the methods where CV1 is the distance between the centre of mass (COM) of backbone atoms of active site residues, along with oxyanion hole forming residues and COM of the substrate. CV2 is the COM distance between the reactive atom of catalytic residue Ser specifically Ser-O and the carboxyl carbon of the substrate.

Sl. no	Simulation	Simulation system contains	CVs applied on	Simulation time (ns)
1	S1	E1 (NAS)	NAS	500
2	S2	EE (NAS + EAS)	NAS	500
3	S3	EE (NAS + EAS)	EAS	500
4	S4	EE (NAS + EAS)	(NAS) and (EAS)	500

### Steered molecular dynamics and umbrella sampling

SMD simulations were conducted on the EE enzyme to delineate the substrate association path from outside to inside where the substrate is exposed to mechanical strain or rupture force, which cannot be achieved through conventional MD simulations. Well-equilibrated systems (after NVT and NPT) were chosen as starting points for the SMD studies^61^. The SMD simulations were implemented using GROMACS 2019.4 tools. The distance between the COM of the substrate and the COM of Ser161 was used to pull the substrate to the NAS. The pull velocity of 0.0005 nm^1^ ps^1^ with the bias force constant of − 80 kJ mol^−1^ nm^−2^ was used. Since very little amount of bias force constant was provided, it is expected to have slow movement of the substrate. The substrate was allowed to move freely in the x, y, and z directions without any constraints (X, Y and Z set to Y Y Y)^62–66^.

Umbrella sampling (US) studies were conducted as an extension of SMD studies to estimate the energetics during the translocation of the substrate in the path of EAS to NAS^67,68^. A series of reaction coordinates across the 17 Å path were chosen from the SMD studies and reaction coordinates or windows were constructed based on the distance between the COM of Ser161 and that of the substrate. The path was discretized into multiple windows for every 0.5 Å of the substrate movement. For umbrella sampling, a total of 34 windows or reaction coordinates covering the 17 Å distance from EAS to NAS were selected, and the simulation was conducted for 10 ns. For statistical significance, SMD and umbrella sampling simulations were run three times^52,69,70^. For the analysis, WHAM Method was used and overlapping windows were removed to obtain a PMF graph^71^.

### Pocket analysis

Tunnels and cavities of varying volumes were identified and analysed using Caver 3.0^72^. Conformations from the trajectory of 500 ns simulation with intervals of 20ps were subjected for the tunnel calculations. The default parameters used for the tunnel calculation were a minimum probe radius of 0.9 Å, and a minimum shell depth of 4 Å shell radius of 3 Å were maintained. The active site was used as the starting point for the origin of the tunnel calculations^49–51^.

### Supplementary Information

Supplementary Figures.

## Data Availability

The datasets used and/or analysed during the current study are available from the corresponding author upon reasonable request.

## Abbreviations

NAS: Natural active site
EAS: Engineered active site
E1: An enzyme with the natural active site
EE: An enzyme with natural and engineered active site
MD: Molecular dynamics
SMD: Steered Molecular Dynamics
QM/MM: Quantum mechanics and molecular mechanics

## References

[1] Reetz, M. T. Lipases as practical biocatalysts. *Curr. Opin. Chem. Biol.* **6**, 145–150. 10.1016/s1367-5931(02)00297-1 (2002). 12038997 10.1016/s1367-5931(02)00297-1

[2] Gopinath, S. C. B., Anbu, P., Lakshmipriya, T. & Hilda, A. Strategies to characterize fungal lipases for applications in medicine and dairy industry. *Biomed. Res. Int.*10.1155/2013/154549 (2013). 23865040 10.1155/2013/154549 PMC3705982

[3] Gupta, R., Gupta, N. & Rathi, P. Bacterial lipases: An overview of production, purification and biochemical properties. *Appl. Microbiol. Biotechnol.* **64**, 763–781. 10.1007/s00253-004-1568-8 (2004). 14966663 10.1007/s00253-004-1568-8

[4] Ortiz, C. *et al.* Novozym 435: The “Perfect” lipase immobilized biocatalyst?. *Catal. Sci. Technol.* **9**, 2380–2420. 10.1039/c9cy00415g (2019). 10.1039/c9cy00415g

[5] Calero, J. *et al.* Selective ethanolysis of sunflower oil with lipozyme RM IM, an immobilized *Rhizomucor miehei* lipase, to obtain a biodiesel-like biofuel, which avoids glycerol production through the monoglyceride formation. *N Biotechnol* **31**(6), 596–601. 10.1016/j.nbt.2014.02.008 (2014). 24594272 10.1016/j.nbt.2014.02.008

[6] Jegannathan, K. R., Abang, S., Poncelet, D., Chan, E. S. & Ravindra, P. Production of biodiesel using immobilized lipase—a critical review. *Critical Rev. Biotechnol.* **28**, 253–264. 10.1080/07388550802428392 (2008). 19051104 10.1080/07388550802428392

[7] Calero, J. *et al.* Selective ethanolysis of sunflower oil with lipozyme RM IM, an immobilized *Rhizomucor miehei* lipase, to obtain a biodiesel-like biofuel, which avoids glycerol production through the monoglyceride formation. *N. Biotechnol.* **31**(6), 596–601. 10.1016/j.nbt.2014.02.008 (2014). 24594272 10.1016/j.nbt.2014.02.008

[8] Park, J. Y. & Park, K. M. Lipase and Its unique selectivity: A mini-review. *J. Chem.*10.1155/2022/7609019 (2022). 10.1155/2022/7609019

[9] Chang, R. C., Chen, J. C. & Shaw, J. F. Studying the active site pocket of staphylococcus hyicuslipase by site-directed mutagenesis. *Biochem. Biophys. Res. Commun.* **229**(1), 6–10 (1996). 8954075 10.1006/bbrc.1996.1749

[10] Sugiura, M. & Isobe, M. Studies on the mechanism of the lipase reaction. *Biochimica et Biophysica Acta (BBA) Enzymol.* **397**(2), 412–417. 10.1016/0005-2744(75)90130-8 (1975). 10.1016/0005-2744(75)90130-8 1171698

[11] Van Der Ent, F. *et al.* Structure and mechanism of a cold-adapted bacterial lipase. *Biochemistry*10.1021/acs.biochem.2c00087 (2022). 35503728 10.1021/acs.biochem.2c00087 PMC9118546

[12] Kumar, A., Dhar, K., Kanwar, S. S. & Arora, P. K. Lipase catalysis in organic solvents: advantages and applications. *Biol. Proced. Online* **18**(1), 2. 10.1186/s12575-016-0033-2 (2016). 26766927 10.1186/s12575-016-0033-2 PMC4711063

[13] Patti, A. & Sanfilippo, C. Stereoselective promiscuous reactions catalyzed by lipases. *Int. J. Mol. Sci.* **23**(5), 2675. 10.3390/ijms23052675 (2022). 35269815 10.3390/ijms23052675 PMC8910291

[14] Sadeghi Googheri, M. S., Housaindokht, M. R. & Sabzyan, H. Reaction mechanism and free energy profile for acylation of candida antarctica lipase B with methylcaprylate and acetylcholine: Density functional theory calculations. *J. Mol. Graph. Model* **54**, 131–140. 10.1016/j.jmgm.2014.10.001 (2014). 25459765 10.1016/j.jmgm.2014.10.001

[15] Santiago, G. *et al.* Rational engineering of multiple active sites in an ester hydrolase. *Biochemistry* **57**(15), 2245–2255. 10.1021/acs.biochem.8b00274 (2018). 29600855 10.1021/acs.biochem.8b00274

[16] Alonso, S. *et al.* Genetically engineered proteins with two active sites for enhanced biocatalysis and synergistic chemo- and biocatalysis. *Nat. Catal.* **3**(3), 319–328. 10.1038/s41929-019-0394-4 (2019). 10.1038/s41929-019-0394-4

[17] Morris, G. M. *et al.* Automated docking using a lamarckian genetic algorithm and an empirical binding free energy function. *J. Comput. Chem.* **19**(14), 16391662 (1998). 10.1002/(SICI)1096-987X(19981115)19:14<1639::AID-JCC10>3.0.CO;2-B

[18] Morris, G. M. *et al.* Software news and updates AutoDock4 and AutoDockTools4: Automated docking with selective receptor flexibility. *J. Comput. Chem.* **30**(16), 2785–2791. 10.1002/jcc.21256 (2009). 19399780 10.1002/jcc.21256 PMC2760638

[19] Hanwell, M. D.; Curtis, D. E.; Lonie, D. C.; Vandermeersch, T.; Zurek, E.; Hutchison, G. R. *SOFTWARE Open Access Avogadro: An Advanced Semantic Chemical Editor, Visualization, and Analysis Platform*; 2012; Vol. 4. https://www.jcheminf.com/content/4/1/17. 10.1186/1758-2946-4-17 PMC3542060 22889332

[20] Forli, S. *et al.* Computational protein-ligand docking and virtual drug screening with the AutoDock suite. *Nat. Protoc.* **11**(5), 905–919. 10.1038/nprot.2016.051 (2016). 27077332 10.1038/nprot.2016.051 PMC4868550

[21] Kumar Ramalingam, P. *et al.* In silico screening of chlorogenic acids from plant sources against human translocase-I to identify competitive inhibitors to treat diabetes. *ACS Omega* **9**(6), 6561–6568. 10.1021/acsomega.3c07267 (2024). 38371776 10.1021/acsomega.3c07267 PMC10870349

[22] Berendsen, H. J. C., van der Spoel, D. & van Drunen, R. GROMACS: A message-passing parallel molecular dynamics implementation. *Comput. Phys. Commun.* **91**(1–3), 43–56. 10.1016/0010-4655(95)00042-E (1995). 10.1016/0010-4655(95)00042-E

[23] Abraham, M. J. *et al.* Gromacs: High performance molecular simulations through multi-level parallelism from laptops to supercomputers. *SoftwareX* **1–2**, 19–25. 10.1016/j.softx.2015.06.001 (2015). 10.1016/j.softx.2015.06.001

[24] Van Der Spoel, D. *et al.* GROMACS: Fast, flexible, and free. *J. Comput. Chem.*10.1002/jcc.20291 (2005). 16211538 10.1002/jcc.20291

[25] Pronk, S. *et al.* GROMACS 4.5: A high-throughput and highly parallel open source molecular simulation toolkit. *Bioinformatics* **29**(7), 845–854. 10.1093/bioinformatics/btt055 (2013). 23407358 10.1093/bioinformatics/btt055 PMC3605599

[26] Lindorff-Larsen, K. *et al.* Improved side-chain torsion potentials for the amber Ff99SB protein force field. *Proteins: Struct. Funct. Bioinform.* **78**(8), 1950–1958. 10.1002/prot.22711 (2010). 10.1002/prot.22711 PMC2970904 20408171

[27] Shabane, P. S., Izadi, S. & Onufriev, A. V. General purpose water model can improve atomistic simulations of intrinsically disordered proteins. *J. Chem. Theory Comput.* **15**(4), 2620–2634. 10.1021/acs.jctc.8b01123 (2019). 30865832 10.1021/acs.jctc.8b01123

[28] Leontyev, I. V; Stuchebrukhov, A. A.; Paragon, A.; Environment, P. *Subscriber Access Provided by UNIV OF ARIZONA Polarizable Mean-Field Model of Water for Biological Simulations Polarizable Mean-Field Model of Water for Biological Simulations with Amber and Charmm Force Fields*; 2012. http://pubs.acs.org. 10.1021/ct300011h PMC4285689 25580096

[29] Anandakrishnan, R., Izadi, S. & Onufriev, A. V. Why computed protein folding landscapes are sensitive to the water model. *J. Chem. Theory Comput.* **15**(1), 625–636. 10.1021/acs.jctc.8b00485 (2019). 30514080 10.1021/acs.jctc.8b00485 PMC11560320

[30] He, X., Man, V. H., Yang, W., Lee, T.-S. & Wang, J. A fast and high-quality charge model for the next generation general AMBER force field. *J. Chem. Phys.*10.1063/5.0019056 (2020). 32962378 10.1063/5.0019056 PMC7728379

[31] Sousa da Silva, A. W. & Vranken, W. F. ACPYPE - AnteChamber PYthon Parser InterfacE. *BMC Res. Notes* **5**(1), 367. 10.1186/1756-0500-5-367 (2012). 22824207 10.1186/1756-0500-5-367 PMC3461484

[32] Zahariev, F., Gordon, M. S. & Levy, M. Energy components in spin-density functional theory. *Phys. Rev. A (Coll Park)* **104**(2), 022815. 10.1103/PhysRevA.104.022815 (2021). 10.1103/PhysRevA.104.022815

[33] Gordon, M. S. & Fischer, H. A molecular orbital study of the isomerization mechanism of diazacumulenes. *J. Am. Chem. Soc.* **90**(10), 2471–2476. 10.1021/ja01012a004 (1968). 10.1021/ja01012a004

[34] Pople, J. A. & Gordon, M. Molecular orbital theory of the electronic structure of organic compounds. I. Substituent effects and dipole moments. *J. Am. Chem. Soc.* **89**(17), 4253–4261. 10.1021/ja00993a001 (1967). 26270792 10.1021/ja00993a001

[35] Harger, M. & Ren, P. Virial-based berendsen barostat on GPUs using AMOEBA in tinker-OpenMM. *Results Chem.*10.1016/j.rechem.2019.100004 (2019). 33868909 10.1016/j.rechem.2019.100004 PMC8049534

[36] Kadoura, A., Salama, A. & Sun, S. Switching between the NVT and NpT ensembles using the reweighting and reconstruction scheme. *Proc. Comput. Sci.* **51**, 1259–1268. 10.1016/j.procs.2015.05.309 (2015). 10.1016/j.procs.2015.05.309

[37] Messias, A., Santos, D. E. S., Pontes, F. J. S., Lima, F. S. & Soares, T. A. Out of sight, out of mind: The effect of the equilibration protocol on the structural ensembles of charged glycolipid bilayers. *Molecules* **25**(21), 5120. 10.3390/molecules25215120 (2020). 33158044 10.3390/molecules25215120 PMC7663769

[38] Wang, Y. L., Zhu, Y. L., Lu, Z. Y. & Laaksonen, A. Electrostatic interactions in soft particle systems: Mesoscale simulations of ionic liquids. *Soft Matter* **14**(21), 4252–4267. 10.1039/c8sm00387d (2018). 29780992 10.1039/c8sm00387d

[39] Abraham, M. J. & Gready, J. E. Optimization of parameters for molecular dynamics simulation using smooth particle-mesh Ewald in GROMACS 4.5. *J. Comput. Chem.* **32**(9), 2031–2040. 10.1002/jcc.21773 (2011). 21469158 10.1002/jcc.21773

[40] Pettersen, E. F. *et al.* UCSF ChimeraX: Structure visualization for researchers, educators, and developers. *Protein Sci.* **30**(1), 70–82. 10.1002/pro.3943 (2021). 32881101 10.1002/pro.3943 PMC7737788

[41] Huang, C. C., Meng, E. C., Morris, J. H., Pettersen, E. F. & Ferrin, T. E. Enhancing UCSF chimera through web services. *Nucleic Acids Res.*10.1093/nar/gku377 (2014). 24861624 10.1093/nar/gku377 PMC4086125

[42] Pettersen, E. F. *et al.* UCSF Chimera—A visualization system for exploratory research and analysis. *J. Comput. Chem.* **25**(13), 1605–1612. 10.1002/jcc.20084 (2004). 15264254 10.1002/jcc.20084

[43] Fernandes, H. S., Sousa, S. F. & Cerqueira, N. M. F. S. A. VMD store-A VMD plugin to browse, discover, and install VMD extensions. *J. Chem. Inf. Model* **59**(11), 4519–4523. 10.1021/acs.jcim.9b00739 (2019). 31682440 10.1021/acs.jcim.9b00739

[44] Humphrey, W., Dalke, A. & Schulten, K. VMD: Visual molecular dynamics. *J. Mol. Graph.* **14**(1), 33–38. 10.1016/0263-7855(96)00018-5 (1996). 8744570 10.1016/0263-7855(96)00018-5

[45] Seeber, M. *et al.* Software news and updates wordom: A user-friendly program for the analysis of molecular structures, trajectories, and free energy surfaces. *J. Comput. Chem.* **32**(6), 1183–1194. 10.1002/jcc.21688 (2011). 21387345 10.1002/jcc.21688 PMC3151548

[46] Seeber, M., Cecchini, M., Rao, F., Settanni, G. & Caflisch, A. Wordom: A program for efficient analysis of molecular dynamics simulations. *Bioinformatics* **23**(19), 2625–2627. 10.1093/bioinformatics/btm378 (2007). 17717034 10.1093/bioinformatics/btm378

[47] Sweeney, P. *et al.* Structure, dynamics, and molecular inhibition of the *Staphylococcus aureus* M1A22-TRNA methyltransferase TrmK. *J. Biol. Chem.* **298**(6), 102040. 10.1016/j.jbc.2022.102040 (2022). 35595101 10.1016/j.jbc.2022.102040 PMC9190014

[48] Sullivan, S. F. *et al.* Towards universal synthetic heterotrophy using a metabolic coordinator. *Metab. Eng.* **79**, 14–26. 10.1016/j.ymben.2023.07.001 (2023). 37406763 10.1016/j.ymben.2023.07.001 PMC10529783

[49] Jurcik, A. *et al.* CAVER Analyst 2.0: Analysis and visualization of Chan–Nels and tunnels in protein structures and molecular dynamics trajectories. *Bioinformatics* **34**(20), 3586–3588. 10.1093/bioinformatics/bty386/4993945 (2018). 29741570 10.1093/bioinformatics/bty386/4993945 PMC6184705

[50] Pavelka, A. *et al.* CAVER: Algorithms for analyzing dynamics of tunnels in macromolecules. *IEEE/ACM Trans. Comput. Biol. Bioinform.* **13**(3), 505–517. 10.1109/TCBB.2015.2459680 (2016). 27295634 10.1109/TCBB.2015.2459680

[51] Kozlikova, B. *et al.* CAVER analyst 1.0: Graphic tool for interactive visualization and analysis of tunnels and channels in protein structures. *Bioinformatics* **30**(18), 2684–2685 (2014). 24876375 10.1093/bioinformatics/btu364

[52] Raju, D. R. *et al.* Extensive modelling and quantum chemical study of sterol C-22 desaturase mechanism: A commercially important cytochrome P450 family. *Catal. Today* **397–399**, 50–62. 10.1016/j.cattod.2021.12.004 (2022). 10.1016/j.cattod.2021.12.004

[53] Bonomi, M.; Camilloni, C. *Biomolecular Simulations Methods and Protocols Methods in Molecular Biology 2022*. http://www.springer.com/series/7651.

[54] Sucerquia, D., Parra, C., Cossio, P. & Lopez-Acevedo, O. Ab initio metadynamics determination of temperature-dependent free-energy landscape in ultrasmall silver clusters. *J. Chem. Phys.* **156**(15), 154301. 10.1063/5.0082332 (2022). 35459298 10.1063/5.0082332

[55] Bonomi, M. *et al.* PLUMED: A portable plugin for free-energy calculations with molecular dynamics. *Comput. Phys. Commun.* **180**(10), 1961–1972. 10.1016/j.cpc.2009.05.011 (2009). 10.1016/j.cpc.2009.05.011

[56] Hsu, W.-T., Piomponi, V., Merz, P. T., Bussi, G. & Shirts, M. R. Alchemical metadynamics: Adding alchemical variables to metadynamics to enhance sampling in free energy calculations. *J. Chem. Theory Comput.* **19**(6), 1805–1817. 10.1021/acs.jctc.2c01258 (2023). 36853624 10.1021/acs.jctc.2c01258

[57] Nava, M. Implementing dimer metadynamics using gromacs. *J. Comput. Chem.* **39**, 2126–2132. 10.1002/jcc.25386 (2018). 30306568 10.1002/jcc.25386

[58] Bertazzo, M., Gobbo, D., Decherchi, S. & Cavalli, A. Machine learning and enhanced sampling simulations for computing the potential of mean force and standard binding free energy. *J. Chem. Theory Comput.* **17**(8), 5287–5300. 10.1021/acs.jctc.1c00177 (2021). 34260233 10.1021/acs.jctc.1c00177 PMC8389529

[59] Shrivastav, G., Khan, T. S., Agarwal, M. & Haider, M. A. A car-parrinello molecular dynamics simulation study of the retro diels-alder reaction for partially saturated 2-pyrones in water. *J. Phys. Chem. C* **122**(22), 11599–11607. 10.1021/acs.jpcc.8b00250 (2018). 10.1021/acs.jpcc.8b00250

[60] Biswas, S.; Wong, B. M. Ab Initio Metadynamics Calculations Reveal Complex Interfacial Effects in Acetic Acid Deprotonation Dynamics; 2021.

[61] Trivedi, V. D. *et al.* In-depth sequence–function characterization reveals multiple pathways to enhance enzymatic activity. *ACS Catal.* **12**(4), 2381–2396. 10.1021/acscatal.1c05508 (2022). 37325394 10.1021/acscatal.1c05508 PMC10270700

[62] Lesitha Jeeva Kumari, J., Jesu Jaya Sudan, R. & Sudandiradoss, C. Evaluation of peptide designing strategy against subunit reassociation in mucin 1: A steered molecular dynamics approach. *PLoS One*10.1371/journal.pone.0183041 (2017). 28817726 10.1371/journal.pone.0183041 PMC5560680

[63] Grubmüller, H., Heymann, B. & Tavan, P. Ligand binding: Molecular mechanics calculation of the streptavidin-biotin rupture force. *Science* **271**(5251), 997–999. 10.1126/science.271.5251.997 (1996). 8584939 10.1126/science.271.5251.997

[64] Skovstrup, S., David, L., Taboureau, O. & Jørgensen, F. S. A steered molecular dynamics study of binding and translocation processes in the GABA transporter. *PLoS One*10.1371/journal.pone.0039360 (2012). 22737235 10.1371/journal.pone.0039360 PMC3380839

[65] Shen, M. *et al.* Steered molecular dynamics simulations on the binding of the appendant structure and helix-Β2 in domain-swapped human cystatin C dimer. *J. Biomol. Struct. Dyn.* **30**(6), 652–661. 10.1080/07391102.2012.689698 (2012). 22731964 10.1080/07391102.2012.689698

[66] Genchev, G. Z. *et al.* Mechanical signaling on the single protein level studied using steered molecular dynamics. *Cell Biochem. Biophys.*10.1007/s12013-009-9064-5 (2009). 19669741 10.1007/s12013-009-9064-5

[67] Vargiu, A. V. *et al.* Water-mediated interactions enable smooth substrate transport in a bacterial efflux pump. *Biochim. Biophys. Acta Gen. Subj.* **1862**(4), 836–845. 10.1016/j.bbagen.2018.01.010 (2018). 29339082 10.1016/j.bbagen.2018.01.010

[68] Bowman, J. D. & Lindert, S. Molecular dynamics and umbrella sampling simulations elucidate differences in troponin C isoform and mutant hydrophobic patch exposure. *J. Phys. Chem. B* **122**(32), 7874–7883. 10.1021/acs.jpcb.8b05435 (2018). 30070845 10.1021/acs.jpcb.8b05435 PMC6098415

[69] Iida, S., Nakamura, H. & Higo, J. Enhanced conformational sampling to visualize a free-energy landscape of protein complex formation. *Biochem. J.*10.1042/BCJ20160053 (2016). 27288028 10.1042/BCJ20160053 PMC4901360

[70] Londhe, A. M. H., Gadhe, C. G., Lim, S. M. & Pae, A. N. Investigation of molecular details of keap1-Nrf2 inhibitors using molecular dynamics and umbrella sampling techniques. *Molecules*10.3390/molecules24224085 (2019). 31726716 10.3390/molecules24224085 PMC6891428

[71] Hub, J. S., de Groot, B. L. & van der Spoel, D. G_wham—a free weighted histogram analysis implementation including robust error and autocorrelation estimates. *J. Chem. Theory Comput.* **6**(12), 3713–3720. 10.1021/ct100494z (2010). 10.1021/ct100494z

[72] Petřek, M. *et al.* CAVER: A new tool to explore routes from protein clefts, pockets and cavities. *BMC Bioinform.*10.1186/1471-2105-7-316 (2006). 10.1186/1471-2105-7-316 PMC1539030 16792811

