# Supplementary material for: Computational studies on the catalytic potential of the double active site for enzyme engineering
Source: Sci Rep. 2024 Aug 2;14:17892. doi: 10.1038/s41598-024-60824-x (PMC11297320; doi:10.1038/s41598-024-60824-x)
Supplement: Supplementary file 1 — Supplementary Figures. [file 41598_2024_60824_MOESM1_ESM.docx]

**SUPPORTING INFORMATION**

**Computational studies on the catalytic potential of the double active site for enzyme engineering**

Naveen Banchallihundi Krishna^1,2^, Lalitha Roopa^1^_,_ Kumar R Pravin*^1^, Gopenath S Thangaraj^2^

^1^Kcat Enzymatic Private Limited, Department of Computational Biology and AI, #16, Ramakrishnappa Road, Cox Town, Bangalore, India 560005.

^2^ Department of Biotechnology & Bioinformatics, JSS Academy of Higher Education & Research, Mysuru-570 015.

**Principle component analysis**

In order to uncover the dominant motions of the E1 and EE, principal component analysis was carried out, and the first three eigenvectors were extracted. In E1 the movement was observed in multiple regions of the protein. E1 shows movements in two regions; the first is the N-terminal region consisting of residues1-35 and the second region consists of residues 190-220. Structurally, the first and second regions are present close to each other. The N-terminal movement could be the cause of dynamic movements of water molecules as the region is exposed. Water molecules are known to be highly dynamic which causes the movement of terminal residues. Since the second region is present close to the N-terminal which is causing the movements. In the case of EE, similar regions show reduced motions in the above-mentioned regions this is because of the presence of EAS between these two regions. The catalytic residues in EAS are Ser211, His214, and Asp25 which are present between above mentioned two regions. The interactions of the substrate with amino acids present in this region reduce the motions. The substrate found to predominantly interact with Met13, Thr17, Gly23, Leu24, Asp25, Ser211, His214, and Trp217.


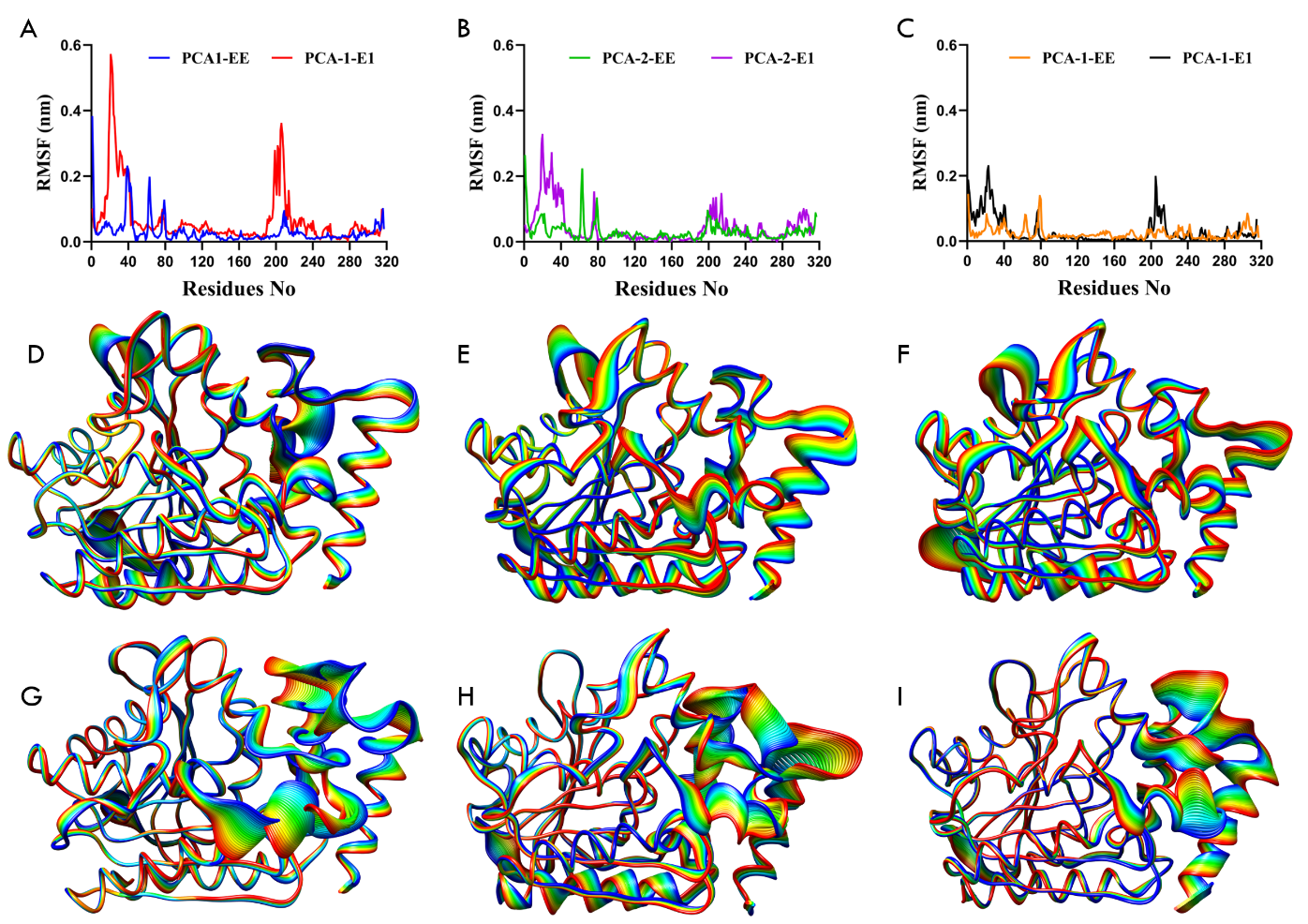


Figure S1: The principal component analysis conducted on 500ns X 3 simulation trajectories. The E1 and EE are both simulated for 500ns X 3 to maintain statistical consistency. A, B, C shows RMSF for E1 and EE. D, E, F and G, H, I show average conformation generated for the first, second and third eigenvectors of E1 and EE respectively. RMSF graph of E1 simulations shows major fluctuations in two regions which are the N-terminal region consisting of residues 1-45 and another region consisting of residues 190-220. Both regions are structurally present close to each other which makes both dynamic.

**Metadynamics:**

The collective variables (CV) are an important component in metadynamics simulations as the free energy is governed by the dynamic behaviour of the collective variables. The CVs for NAS and EAS were explained structurally in the below figure.

**
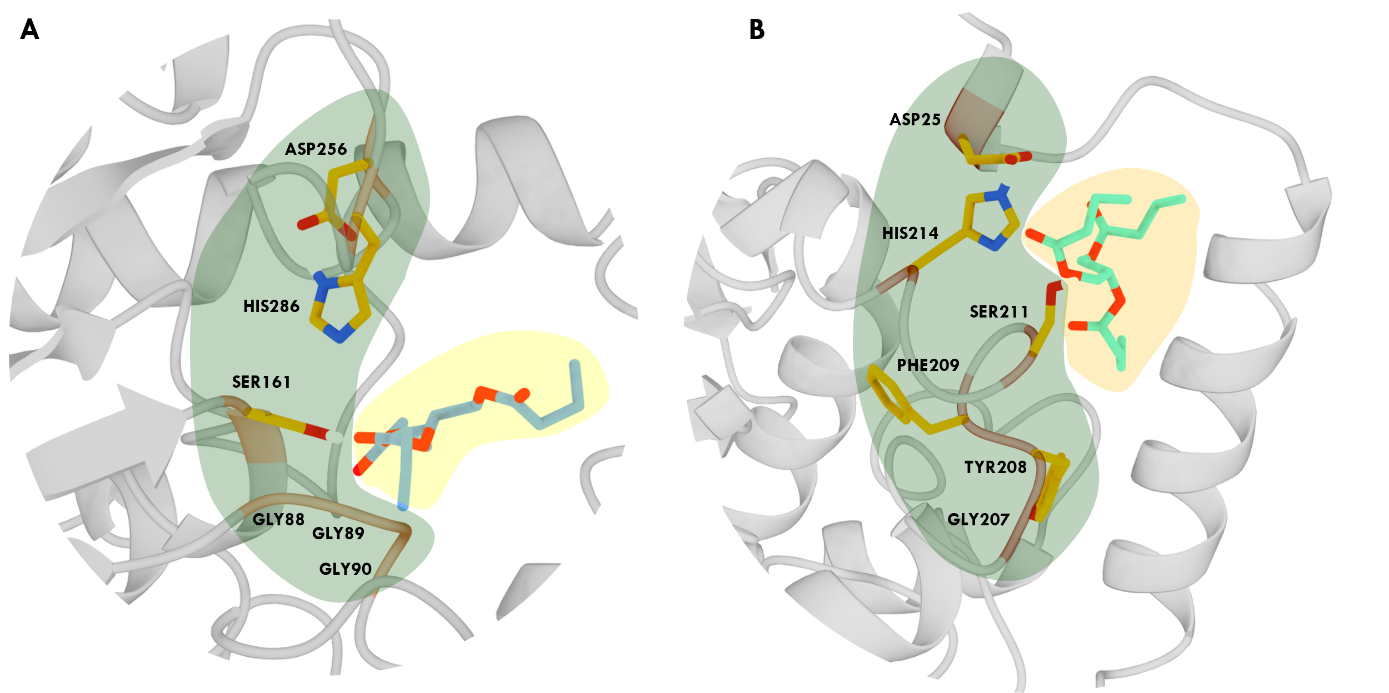
**

Figure S2: The figure shows CVs generated for Metadynamics simulations. A) the CV constructed for E1 simulation where NAS active site and substrate present. B) The CV constructed for EE where the EAS active site is present. For metadynamics simulations, CVs play an important role in finding the least energy conformation across the active site. The applied bias potential allows substrate molecules to find different minima all around the active site which is not possible through regular molecular dynamics. Here for NAS and EAS in E1 and EE respectively, CV1 is the distance between the centre of mass (COM) of backbone atoms of active site residues, along with oxyanion hole forming residues (Green bubble) and COM of the substrate (yellow bubble). CV2 is the COM distance between the reactive atom of catalytic residue Ser specifically Ser-O and the carboxyl carbon of the substrate.
